# Knock-In Mice Expressing a 15-Lipoxygenating Alox5 Mutant Respond Differently to Experimental Inflammation Than Reported *Alox5^−/−^* Mice

**DOI:** 10.3390/metabo11100698

**Published:** 2021-10-12

**Authors:** Eugenia Marbach-Breitrück, Nadine Rohwer, Carmen Infante-Duarte, Silvina Romero-Suarez, Dominika Labuz, Halina Machelska, Laura Kutzner, Nils Helge Schebb, Michael Rothe, Pallu Reddanna, Karsten H. Weylandt, Lothar H. Wieler, Dagmar Heydeck, Hartmut Kuhn

**Affiliations:** 1 Department of Biochemistry, Charité-Universitätsmedizin Berlin, Corporate Member of Freie Universität Berlin and Humboldt-Universität zu Berlin, Chariteplatz 1, 10117 Berlin, Germany; eugenia.marbach@yahoo.de (E.M.-B.); dagmar.heydeck@charite.de (D.H.); 2 Division of Medicine, Department of Gastroenterology, Metabolism and Oncology, Ruppin General Hospital, Brandenburg Medical School, Fehrbelliner Straße 38, 16816 Neuruppin, Germany; nadine.rohwer@mhb-fontane.de (N.R.); karsten.weylandt@mhb-fontane.de (K.H.W.); 3 Faculty of Health Sciences, Joint Faculty of the Brandenburg University of Technology Cottbus-Senftenberg, Brandenburg Medical School and University of Potsdam, 14469 Potsdam, Germany; 4 Department of Hepatology, Charité-Universitätsmedizin Berlin, Corporate Member of Freie Universität Berlin and Humboldt-Universität zu Berlin, Gastroenterology and Metabolism, Augustenburger Platz 1, 13353 Berlin, Germany; 5 Department of Molecular Toxicology, German Institute of Human Nutrition Potsdam-Rehbruecke, Arthur-Scheunert-Allee 114-116, 14558 Nuthetal, Germany; 6 Institute for Medical Immunology, Charité-Universitätsmedizin Berlin, Corporate Member of Freie Universität Berlin and Humboldt-Universität zu Berlin, Augustenburger Platz 1, 13353 Berlin, Germany; carmen.infante@charite.de (C.I.-D.); silvina.romero-suarez@medma.uni-heidelberg.de (S.R.-S.); 7 Department of Experimental Anesthesiology, Charité-Universitätsmedizin Berlin, Corporate Member of Freie Universität Berlin and Humboldt-Universität zu Berlin, Hindenburgdamm 30, 12203 Berlin, Germany; dominika.labuz@charite.de (D.L.); hmachelska@gmail.com (H.M.); 8 Chair of Food Chemistry, Faculty of Mathematics and Natural Sciences, University of Wuppertal, Gaussstraße 20, 42119 Wuppertal, Germany; laura.kutzner@schebb-web.de (L.K.); nils@schebb-web.de (N.H.S.); 9 Lipidomix GmbH, Robert-Rössle-Straße 10, 13125 Berlin, Germany; michael.rothe@lipidomix.de; 10 Department of Animal Biology, School of Life Sciences, University of Hyderabad, Gachibowli, Hyderabad 500046, Andhra Pradesh, India; preddanna@gmail.com; 11 Robert Koch Institute, Nordufer 20, 13353 Berlin, Germany; WielerLH@rki.de; 12 Center of Infection Medicine, Institute of Microbiology and Epizootics, Free University of Berlin, Robert-von-Ostertag-Str. 7-13, 14163 Berlin, Germany

**Keywords:** eicosanoids, lipoxygenase, inflammation, pain, leukotrienes, resolvins

## Abstract

Arachidonic acid 5-lipoxygenase (ALOX5) is the key enzyme in the biosynthesis of pro-inflammatory leukotrienes. We recently created knock-in mice (*Alox5*-KI) which express an arachidonic acid 15-lipoxygenating Alox5 mutant instead of the 5-lipoxygenating wildtype enzyme. These mice were leukotriene deficient but exhibited an elevated linoleic acid oxygenase activity. Here we characterized the polyenoic fatty acid metabolism of these mice in more detail and tested the animals in three different experimental inflammation models. In experimental autoimmune encephalomyelitis (EAE), *Alox5*-KI mice displayed an earlier disease onset and a significantly higher cumulative incidence rate than wildtype controls but the clinical score kinetics were not significantly different. In dextran sodium sulfate-induced colitis (DSS) and in the chronic constriction nerve injury model (CCI), *Alox5*-KI mice performed like wildtype controls with similar genetic background. These results were somewhat surprising since in previous loss-of-function studies targeting leukotriene biosynthesis (*Alox5*^−/−^ mice, inhibitor studies), more severe inflammatory symptoms were observed in the EAE model but the degree of inflammation in DSS colitis was attenuated. Taken together, our data indicate that these mutant *Alox5*-KI mice respond differently in two models of experimental inflammation than *Alox5^−/−^* animals tested previously in similar experimental setups.

## 1. Introduction

The arachidonic acid lipoxygenases (ALOX-isoforms) are lipid-peroxidizing enzymes [1,2], which have been implicated in the biosynthesis of pro- and anti-inflammatory mediators [3,4]. The human genome involves six functional ALOX genes (*ALOX15, ALOX15B, ALOX12, ALOX12B, ALOXE3, ALOX5*), most of which are localized in the joint ALOX-gene cluster on chromosome 17 [5]. In contrast, the *ALOX5* gene was mapped to chromosome 10. In the mouse genome a single ortholog exists for each human gene, but in addition a functional *Aloxe12* gene is present in the *Alox* gene cluster on mouse chromosome 17 [5].

Mammalian ALOX5 orthologs are essential for the biosynthesis of pro-inflammatory leukotrienes [3], and the first ALOX5 ortholog was discovered in leukocytes of different species [6]. *ALOX5* genes are present in the genome of most mammals, but have also been described in lower vertebrates, invertebrates and metazoa [7,8]. ALOX5 orthologs catalyze the conversion of arachidonic acid to 5,6-epoxyleukotriene (LTA_4_). This unstable intermediate is further hydrolyzed by LTA_4_ hydrolase [9] to leukotriene B_4_ (LTB_4_) or is alternatively converted by LTC_4_ synthase [10] to leukotriene C_4_ (LTC_4_). LTB_4_ is a strong pro-inflammatory mediator, which activates neutrophils but also induces chemokinesis of inflammatory cells [11,12]. LTC_4_ and LTD_4_ induce sustained bronchoconstriction, bronchial mucus secretion and increased permeability of the blood vessel wall [13]. Currently, cysteinyl leukotriene receptor antagonists [14] and a specific ALOX5 inhibitor [15] are available for prescription for the treatment of bronchial asthma. *Alox5*-deficient mice were protected from inflammation in different animal disease models [16], and administration of a lipid-rich Western diet to these animals induced the formation of aortic aneurisms [17].

ALOX15 was first discovered in rabbit reticulocytes [18]. The enzyme is expressed as cytosolic protein and was purified to homogeneity in mg amounts [19]. The properties of rabbit ALOX15 [19] and of other mammalian ALOX15 orthologs [20,21,22] have been characterized and the enzymes exhibit two catalytic peculiarities: (i) They show dual reaction specificity with arachidonic acid [23,24]; (ii) they oxygenate esterified polyenoic fatty acids even if these substrates are present in ester lipids of lipoproteins and biomembranes [18]. *Alox15*^−/−^ mice are viable and grow normally [25] but they exhibit significantly reduced erythrocyte counts and subtle reticulocytosis [26]. In different murine inflammation models, *Alox15^−/−^* mice develop more [27] or less [28] intense inflammatory symptoms, and thus ALOX15 may play a regulatory role in inflammation. The anti-inflammatory activity of ALOX15 has been related to the involvement of this enzyme in the biosynthesis of pro-resolving mediators [29] or in the activation cascade of the peroxisome proliferation activating receptor gamma (PPARγ) [30].

The inverse biological functions of ALOX5 (pro-inflammatory) and ALOX15 (pro-resolving) are directly related to the reaction specificity of the two enzymes. Arachidonic acid 5-lipoxygenation is essential for the biosynthesis of pro-inflammatory leukotrienes [1], whereas arachidonic acid 15-lipoxygenation has been implicated in the biosynthesis of specialized pro-resolving mediators [4,29]. If one could alter the reaction specificity of ALOX5 from arachidonic acid 5- to arachidonic acid 15-lipoxygenation, it should be possible to transform the pro-inflammatory ALOX5 to a pro-resolving enzyme. It should be stressed at this point that the idea that mammalian ALOX5 orthologs are pro-inflammatory enzymes and that ALOX15 orthologs are anti-inflammatory is rather simplistic. For instance, the biosynthesis of specialized pro-resolving mediators (SPMs) may involve both ALOX5 and ALOX15 orthologs [4,31]. Moreover, there are ALOX5-dependent and ALOX5-independent biosynthetic pathways for the formation of SPMs [4,31]. Because of this complexity, it is impossible to predict the impact of selective functional modification of the *ALOX5* gene on the biosynthetic capacity of inflammatory cells to produce SPMs.

Several years ago, we reported that combined mutations of the triad determinants of human ALOX5 (Phe359Trp + Ala424Ile + Asn425Met + Ala603Ile) induced such a switch in the reaction specificity [32], and similar specificity alterations were later observed for mouse Alox5 [33]. To explore whether this mutagenesis concept also works in vivo, we recently created knock-in mice, which express the Alox5 Phe359Trp + Ala424Ile + Asn425Met triple mutant instead of the wildtype enzyme [34]. These animals exhibit defective leukotriene biosynthesis [34], but it has not been explored how they perform under inflammatory conditions.

To fill this gap, we tested our *Alox5* knock-in (*Alox5-*KI) mice in three different animal disease models associated with inflammation. We found that in the experimental autoimmune encephalomyelitis (EAE) model and in the dextran sodium sulfate induced colitis (DSS) model, *Alox5*-KI mice showed different responses than other leukotriene-deficient animals explored in the literature [35,36].

## 2. Results

### 2.1. Functional Characterization of Alox5 Knock-in Mice (Alox5-KI)

In our *Alox5*-KI mice, the *Alox5* gene was modified by site-directed mutagenesis of the triad determinants to reach two independent goals: i) Inactivation of the arachidonic acid 5-lipoxygenase activity and the leukotriene synthase activity of Alox5. As the first consequence of this alteration, we expected that the *Alox5*-KI mice might perform like *Alox5*^−/−^ mice in animal disease models, in which leukotrienes may play a major patho-physiological role. ii) Upregulation of the arachidonic acid 15-lipoxygenating activity. As a second consequence of this alteration, we expected an improved formation of 13-hydroxy-linoleic acid (13-HODE) from linoleic acid and 15-hydroxy arachidonic acid (HETE) from arachidonic acid). The second consequence may overlay the biological effects induced by leukotriene deficiency.

**Figure 1:**
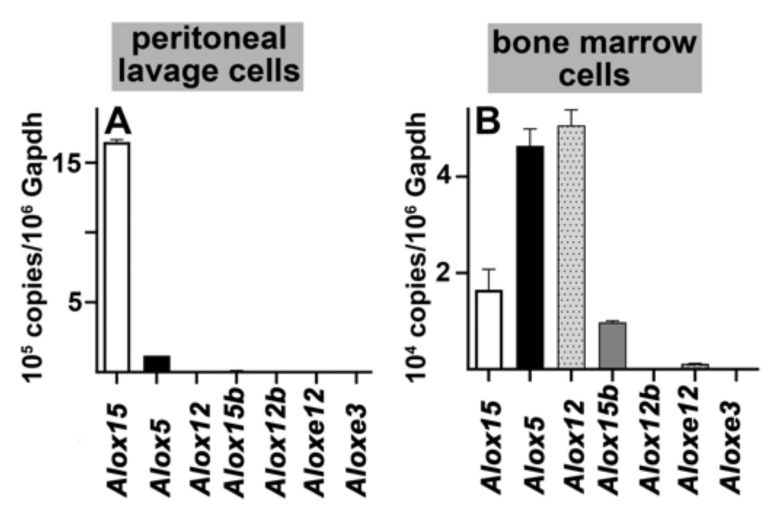
Expression of Alox mRNAs in peritoneal lavage and bone marrow cells of wildtype mice. Cells were prepared from wildtype mice as described in the Materials and Methods section. Total RNA was extracted, reversely transcribed and the different Alox mRNA species were quantified by qRT-PCR using external amplification standards for each target mRNA (mRNAs of different Alox paralogs) and the reference mRNA (Gapdh). (**A**) qRT-PCR of mouse Alox mRNAs of wildtype peritoneal lavage cells. For this experiment the RNA of the cell preparation obtained from four different animals (*n* = 4) was pooled and three independent PCR runs were carried out. (**B**) qRT-PCR of mouse Alox mRNAs of wildtype bone marrow cells. For this experiment, three independent PCR runs were carried out with each RNA pool (*n* = 4).

Before using these animals in the different inflammatory disease models, we first characterized the functional alterations induced by this genetic manipulation. With some exceptions, ALOX isoforms are either expressed in myeloid cells (ALOX15, ALOX15B, ALOX12, ALOX5) or in the skin (ALOX12B, ALOXE3, ALOXE12). Since we were mainly interested in the myeloid ALOX isoforms, we selected peritoneal lavage cells and bone marrow cells as targets for the functional characterization studies. As solid organs, peritoneal lavage and bone marrow cells are heterogenous mixtures of different cell types, but abundant expression of different ALOX-isoforms in these cell populations is well documented. In the first step, we quantified by qRT-PCR the mRNA expression levels of all 7 mouse Alox-isoforms in these cells of wildtype mice and found (Figure 1A) that *Alox15* mRNA is dominantly present in peritoneal lavage cells. *Alox5* mRNA was detected in much smaller amounts, but the mRNAs of the other isoforms were virtually absent. In bone marrow cells (Figure 1B), the situation was more complex. Here we observed that *Alox12* mRNA was most abundant, followed by the mRNAs of *Alox5*, *Alox15* and *Alox15b*. The mRNAs of epidermal Alox isoforms (Alox12b, Aloxe3, Aloxe12) were only detected at very low steady state mRNA concentrations.

In a previous study, the expression levels of other Alox-isoforms were different when *Alox5*-KI mice were compared with corresponding wildtype controls [34]. In fact, comparison of the steady state concentrations of the mRNAs of Alox15 and Alox12 in bone marrow cells of mice did not reveal significant differences between the two genotypes. Thus, our genetic manipulation of the *Alox5* gene does not induce a compensatory upregulation in the expression of other Alox isoforms.

**Figure 2:**
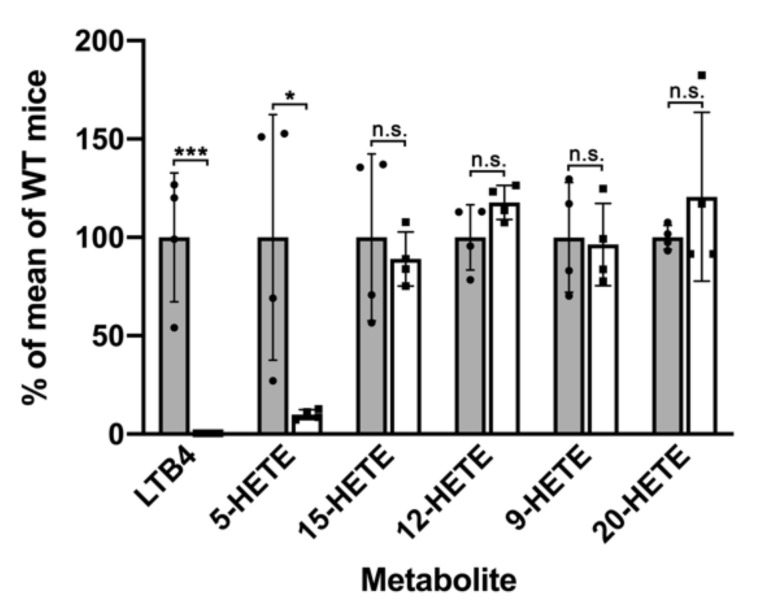
Alox5 ex vivo activity assays using whole blood. *Alox5*-KI mice and corresponding outbred wildtype controls (*n* = 4 of each genotype) were sacrificed under anesthesia and heparinized blood was drawn. Whole blood was incubated for 30 min at 37 °C with 50 μM calcium ionophoreA23187, cells were spun down and eicosanoid profiles were quantified by LC-MS/MS, as described in the Materials and Methods. For each product, the mean of metabolite formation of wildtype mice was set at 100% and product formation of each individual of either genotype was normalized to this value. Gray columns show wildtype mice, white columns show *Alox5*-KI mice. genotype was normalized to this value. Gray columns show wildtype mice, white columns show *Alox5*-KI mice. Dots indicate the individual values for wildtype mice, square indicate the individual values for *Alox5*-KI mice. Statistical analyses comparing *Alox5-*KI mice with wildtype animals were carried out with the two-sided Students *t*-test and the levels of statistical significance are indicated by asterixis (*). * *p* < 0.05, *** *p* < 0.001; n.s. indicates no significant differences between the two genotypes. Data are expressed as means ± SEM, *n* = 4 for each genotype.

To provide additional evidence for the leukotriene deficiency of our *Alox5*-KI mice, we carried out ex vivo activity assays using peripheral blood as an enzyme source. For this purpose, heparinized blood was drawn from *Alox5*-KI mice and corresponding outbred wildtype control animals (WT) and incubated for 30 min in the presence of 50 μM calcium ionophore A23187. Blood cells were spun down and the profiles of relevant eicosanoids in the blood plasma were quantified by LC-MS/MS (see Materials and Methods). From Figure 2, it can be seen that after ionophore stimulation significant amounts of leukotriene B_4_ (LTB_4_) were present in the blood plasma of WT animals. In contrast, no LTB_4_ could be detected when the blood plasma of *Alox5*-KI mice was analyzed. These data indicate that our *Alox5*-KI mice are leukotriene deficient. Simultaneously, we analyzed the plasma levels of 5-HETE and found significantly smaller quantities of this eicosanoid in the blood plasma of *Alox5*-KI mice (Figure 2) when compared with WT controls. Since 5-HETE can also be formed via ALOX5-independent pathways, it is plausible that we did not observe complete abolishment of 5-HETE formation. Our knock-in strategy converted the mouse Alox5 to an arachidonic acid 15-lipoxygenating enzyme and thus it was expected that the 15-HETE levels in the *Alox5*-KI mice should be elevated. Unfortunately, in our lipidomic analyses we did not find increased 15-HETE levels in the plasma lipids (Figure 2). The most plausible explanation for these data is that the amounts of 15-HETE formed by the mutant Alox5 are not high enough to increase the systemic 15-HETE plasma levels. Finally, we quantified the plasma levels of other HETE-isomers (12-HETE, 9-HETE, 20-HETE) but did not observe significant differences between *Alox5*-KI mice and wildtype controls.

**Figure 3:**
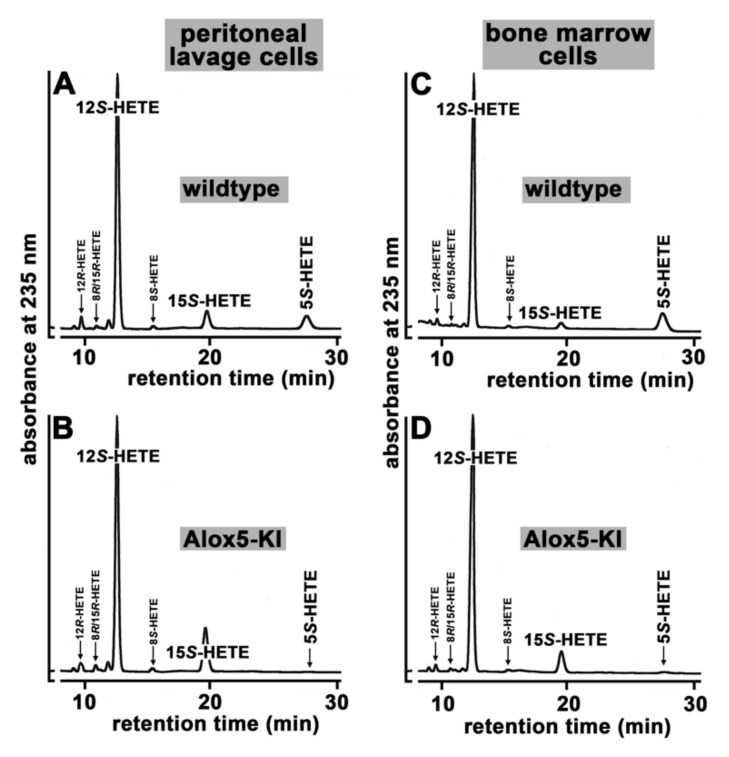
Analysis of the Alox products formed during ex vivo activity assays of peritoneal lavage cells and bone marrow cells of *Alox5*-KI and wildtype mice. Cells were prepared and ex vivo activity assays were carried out (see Materials and Methods). The conjugated dienes formed during the 15 min incubation period were prepared by RP-HPLC and further analyzed by NP/CP-HPLC (see Materials and Methods). Each chromatogram was scaled for the 12*S*-HETE peak. Four independent individuals (*n* = 4) were analyzed for each genotype. Representative partial chromatograms are shown, and statistical evaluation of the product patterns is given in Table 1. (**A**) Wildtype peritoneal lavage cells. (**B**) Peritoneal lavage cells prepared from *Alox5*-KI mice. (**C**) Wildtype bone marrow cells. (**D**) Bone marrow cells prepared from *Alox5*-KI mice.

Since the *Alox5* gene in mice is expressed at high levels in peritoneal lavage cells and in bone marrow cells (Figure 1A,B), we carried out comparative ex vivo activity assays with these cell types prepared from *Alox5*-KI mice and outbred wildtype controls. For these experiments, the activity assay was optimized for ALOX5 measurements (addition of ionophore A23187) and we added ATP, EDTA, Ca^2+^ and phosphatidylcholine to the assay mixture. To test the chirality of the major reaction products we employed NP/CP-HPLC with UV-detection as an analytical method. Under these conditions, peritoneal lavage cells of wildtype controls produce 12*S*-HETE as major product, but smaller amounts of 15*S*-HETE and 5*S*-HETE were also detected (Figure 3A).

When peritoneal lavage cells of *Alox5*-KI mice (Figure 3B) were used, the relative share of 15*S*-HETE formation was significantly elevated (8.1 ± 0.4 % for wildtype cells vs. 18.0 ± 2.4 % for *Alox5*-KI cells, *p* < 0.001, two-sided *t*-test) but 5*S*-HETE formation was almost completely abolished (Figure 3B, Table 1). When wildtype bone marrow cells were employed (Figure 3C), 12*S*-HETE was also the major arachidonic acid (AA) oxygenation product. As with peritoneal lavage cells, small amounts of 5*S*- and 15*S*-HETE were also produced and the relative shares of these Alox products are quantified in Table 1. As expected, corresponding cells of *Alox*5-KI mice did not produce major amounts of 5*S*-HETE (Figure 3D). On the other hand (Figure 3D), the relative share of 15*S*-HETE formation increased five-fold (2.2 ± 0.5% for wildtype bone marrow cells vs. 10.3 ± 0.4% for *Alox5*-KI bone marrow cells, *p* < 0.001, two-sided *t*-test). These alterations in the product pattern could be predicted from our genetic manipulation. It should be stressed at this point that trace amounts of 5-HETE (minor peaks in Figure 3B,D) were also detected in the product mixture formed by bone marrow cells and peritoneal lavage cells of *Alox5*-KI mice. Such trace amounts were detected in the no-cell control incubations since HETE isomers were present in small amounts in the arachidonic acid solution we used as substrate for the ex vivo activity assays. Thus, the very small amounts of 5-HETE detected in the incubation samples most probably originate from the impurities of the arachidonic acid solution used as substrate for the ex vivo activity assays but not from the residual AA 5-lipoxygenating activity of the mutated Alox5.

**Figure 4:**
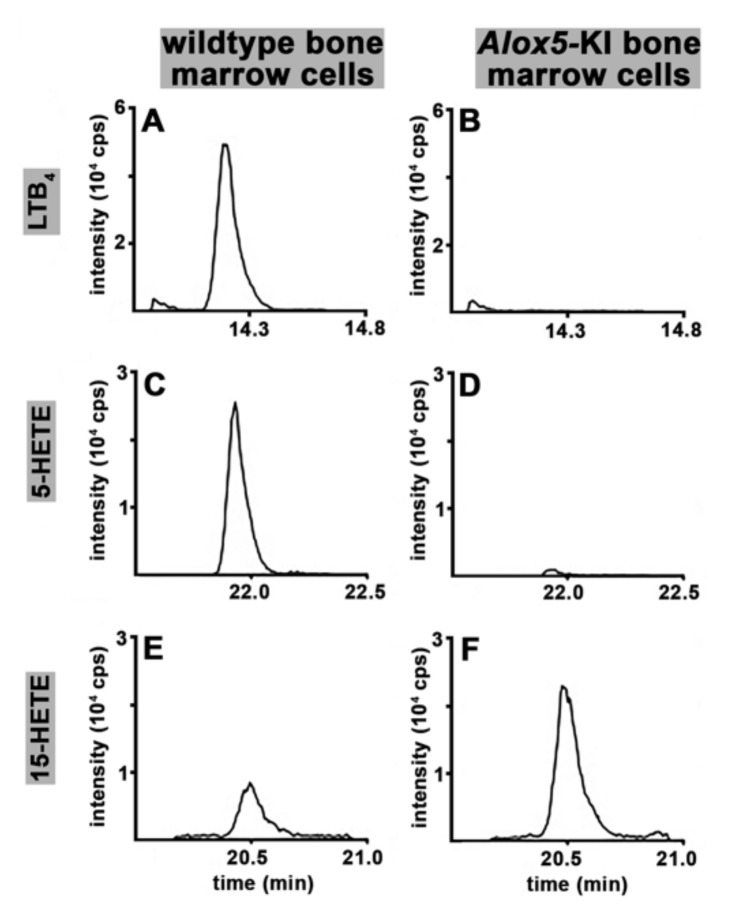
Ex vivo Alox activity assays of bone marrow cells prepared from *Alox5*-KI mice and corresponding wildtype controls using endogenous substrates. Pooled bone marrow cells of either *Alox5*-KI mice (*n* = 5; panels **B**,**D** and **F**) or WT-mice (*n* = 3, panels **A**,**C** and **E**) were used. For each assay 2 × 10^7^ cells were reconstituted in 0.5 mL PBS and were incubated at room temperature in the presence of 5 μM calcium ionophore A23187 for 5 min. Total lipids were extracted (ethyl acetate), solvent was evaporated, the remaining lipids were reconstituted in 50 μL methanol and analyzed for LTB_4_ (panels **A**,**B**), 5-HETE (panels **C**,**D**) and 15-HETE (panels **E,F**) by LC-MS/MS. Chromatographic separation of lipid extracts was carried out using a Zorbax Eclipse Plus C18 RP-column (Agilent, Waldbronn, Germany) and for MS/MS detection the QTRAP instrument (Sciex, Darmstadt, Germany) was operated in negative electrospray ionisation mode. Shown are SRM traces of cell extracts for LTB_4_ (*m/z* 335.2 → 195.1), 5-HETE (*m/z* 319.2 → 115.2) and 15-HETE (*m/z* 319.2 → 219.2).

To explore whether bone marrow cells of *Alox5*-KI mice also exhibit a compromised leukotriene synthesizing capacity using endogenous substrate, we stimulated these cells ex vivo with calcium ionophore and analyzed the formation of leukotriene B_4_, 5-HETE and 15-HETE using an LC-MS/MS based method [37]. Wildtype bone marrow cells (Figure 4A) produced significant amounts of LTB_4_. As expected, when the activity assay was carried out in the presence of 10 μM of the specific ALOX5 inhibitor zileuton [15,38], LTB_4_ formation was completely abolished (Figure S1). Moreover, *Alox5*-KI bone marrow cells did not produce LTB_4_ (Figure 4B). Next, we analyzed the formation of 5-HETE in this ex vivo activity assay and obtained identical results. Large amounts of 5-HETE were formed by wildtype bone marrow cells (Figure 4C) but only traces of this metabolite were detected when *Alox5*-KI bone marrow cells were employed (Figure 4D). These trace amounts of 5-HETE most likely originate from non-enzymatic lipid peroxidation of the liberated arachidonic acid. When cells are stimulated with calcium ionophore, arachidonic acid is released from the cellular membranes and most of this endogenous substrate is oxygenated via the Alox5 pathway. However, small amounts of the liberated substrate may undergo Alox5-independent lipid peroxidation and this share may even be augmented when Alox5-dependent 5-HETE formation is prevented.

Finally, we explored the formation of 15-HETE from endogenous substrates. Here we found that small amounts of this metabolite were formed by wildtype bone marrow cells (Figure 4E). However, *Alox5*-KI bone marrow cells produced more than twice as much 15-HETE (Figure 4F). When the activity assays were carried out in the presence of 10 μM zileuton, 15-HETE formation was reduced to near wildtype levels (Figure S2). The most plausible explanation for these results is that the mutant Alox5 is responsible for the increased 15-HETE synthesizing capacity of *Alox5*-KI bone marrow cells.

### 2.2. Alox5-KI Mice Show Earlier Disease Onset and Higher Cumulative Incidence Rates Than Wildtype Animals in the EAE Model

The EAE model is a frequently employed murine system to explore the patho-physiological mechanisms of human demyelinating diseases [39], and several inhibitors of the ALOX pathway attenuated the severity of clinical symptoms in this model [40,41,42]. Moreover, microarray analyses identified ALOX5 as a component of inflammatory brain lesions in human multiple sclerosis and in the EAE mouse model [43]. These data suggested that leukotriene deficiency should lead to attenuated neurological symptoms. In contrast, the symptoms were exacerbated in *Alox5*^−/−^ mice [35]. In our *Alox5*-KI mice, two principle functional alterations were combined: (i) Lack of leukotriene biosynthesis, which should lead to reduction of the inflammatory symptoms [16], (ii) augmented capacity for the formation of 15-HETE and 13-HODE, which may activate PPARgamma signaling and may suppress inflammation [30]. Thus, based on these previous experimental data it was impossible to predict how the *Alox5-*KI mice might perform in the EAE model.

**Figure 5:**
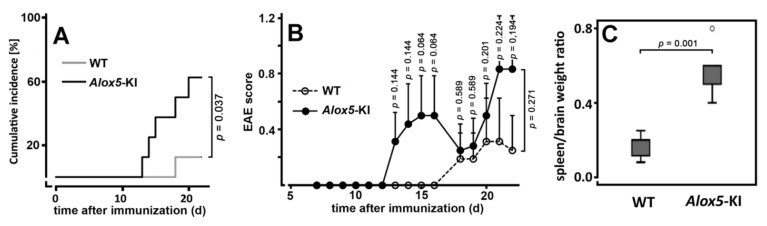
Comparison of *Alox5*-KI and wildtype mice in the EAE inflammation model. *Alox5*-KI mice and wildtype controls (*n* = 8 in each group) were taken through the experimental protocol (see Materials and Methods). The clinical EAE scores were determined at the time points indicated. At the end of the experiment the animals were sacrificed at day 22 of the experimental protocol. Data were statistically evaluated using Mann-Whitney U-Test and the mixed ANOVA approach was used to compare the EAE score kinetics of the two genotypes over time. Cumulative incidence was statistically analyzed by Log-Rank test. (**A**) Onset of disease as indicated by the first appearance of neurological symptoms. (**B**) EAE score kinetics. Data are means ± SEM. (**C**) Relative spleen weight vs. brain weight ratios of the two genotypes (*n* = 8 in each experimental group) at the end of the experimental protocol (day 22).

To answer this question, we induced a mild form of EAE in *Alox5*-KI mice and wildtype controls with identical genetic backgrounds and monitored the neurological symptoms over a time period of 22 days. As intended, under our experimental conditions the incidence of clinically manifested EAE was rather low in wildtype mice since seven out of eight animals did not develop neurological abnormalities (Figure 5A). On the other hand, five of the eight *Alox5*-KI mice showed neurological defects. In fact, the cumulative incidence rate of *Alox5*-KI mice was significantly (*p* = 0.037) higher than that of the wildtype controls (Figure 5A). Next, we quantified the EAE scores for all mice and evaluated these data statistically. Here we found (Figure 5B) that in wildtype mice only a minor increase in the EAE score was observed. In contrast, *Alox5*-KI mice developed more pronounced disease symptoms and a biphasic time course became evident. For these mice, the mean EAE scores strongly increased between days 13 and 16, but at day 18 the scores declined again. This attenuation was related to the partial recovery of two animals from their neurological symptoms. However, at later time points these animals became sick again, which contributed to the further raise of the mean EAE score at later time points. It should be stressed at this point that despite the biphasic disease kinetics, the means of the EAE scores of the *Alox5*-KI mice were always higher than those of the wildtype animals during the entire time course of the experiment. However, when we compared the two EAE score kinetics using the mixed ANOVA test we did not observe significant differences between the two genotypes. Moreover, when we compared the EAE scores for the two genotypes at each time point of the disease we never observed significant differences. Thus, in vivo Phe359Trp + Ala424Ile + Asn425Met triple mutation of the *Alox5* gene does not significantly alter the responsiveness of mice in the EAE model, although a trend for an increased responsiveness was observed. Finally, we quantified different lymphocyte populations in the peripheral blood of wildtype mice and *Alox5*-KI animals at the end of the experimental protocol (Figure S3). However, we did not detect significant differences between the two genotypes (*n* = 3 per group).

When we sacrificed the animals at the end of the experiments, we observed that the spleens of the *Alox5*-KI mice were enlarged. When we normalized the spleen weight to the brain weight of each animal (calculation of the spleen weight/brain weight ratios), we found that this ratio was significantly higher in *Alox5*-KI mice when compared with wildtype controls (Figure 5C). It remains unclear for the moment why the *Alox5*-KI mice develop splenomegaly when taken through this experimental protocol.

Taken together, these data indicated that *Alox5*-KI mice do not behave like *Alox5^−/−^* mice [35] in the EAE model despite the fact that both types of animals were unable to biosynthesize leukotrienes. Thus, the second functional consequence of our genetic manipulation (augmented 15-HETE and 13-HODE formation) may play a modulating role in this animal model of inflammation (see Discussion).

### 2.3. Alox5-KI Mice Are Not Protected from Inflammation in the DSS Colitis Model

The DSS colitis is a frequently employed animal disease model mirroring the patho-physiology of human inflammatory bowel diseases [44]. Previous experiments with the clinically used ALOX5 inhibitor zileuton suggested that inactivation of endogenous leukotriene biosynthesis attenuated the inflammatory symptoms in different animal colitis models [36,45].

**Figure 6:**
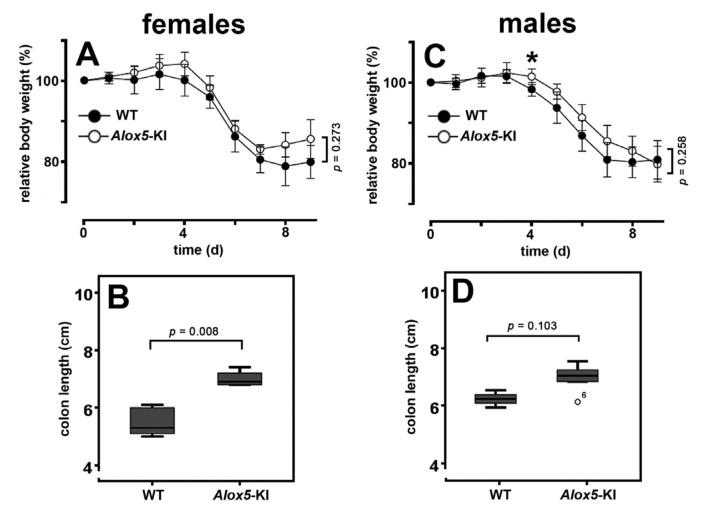
Comparison of *Alox5*-KI mice and wildtype controls in the DSS colitis model. *Alox5*-KI mice (5 males, 5 females) and outbred wildtype controls (5 males, 5 females) were taken through the experimental protocol (see Materials and Methods), and we determined two major clinical readout parameters (body weight, colon length). (**A**,**C**) Kinetics of the relative weight loss that were calculated as percentage of the body weight at the time of measurement related to the body weight of the corresponding individual before the onset of DSS treatment. At each time point data were statistically evaluated using the Mann-Whitney U-Test and mixed ANOVA approach was used to compare weight loss kinetics of the two genotypes over time. * means there are no significant differences. Data are expressed as means ± SEM. (**B,D**) Colon lengths at the end of the experimental protocol. After the animals were sacrificed at the end of the experimental protocol (day 9), the colons were prepared and their lengths were determined.

When we induced DSS colitis in our *Alox5*-KI mice and wildtype controls we only observed a minor difference between the two genotypes. Using the relative loss in body weight as major clinical readout parameter, we found that at most time points the means of *Alox5*-KI were higher than those of wildtype controls (Figure 6A,C). However, these differences did not reach the level of statistical significance at most time points (Mann-Whitney U-test). Comparing the complete body weight kinetics for either female or male individuals (mixed ANOVA), we did not observe significant differences. Finally, we quantified the colon lengths at the end of the experiment as independent readout parameter for colitis severity. DSS-induced colitis leads to colon shrinkage and the degree of colon shortening mirrors the severity of the disease [44]. When we compared the average colon length of DSS-treated wildtype mice with that of *Alox5*-KI animals we observed a significant difference (*p* = 0.008) for female individuals (Figure 6B). For males (Figure 6D) we also observed colon shrinkage, but the difference between the two genotypes did not reach the level of statistical significance (*p* = 0.103). At the end of the experiment, we quantified the disease activity index (DAI) as the third clinical parameter of colitis severity. Here, we did not observe significant differences for males (*p* > 0.940, Mann-Whitney U-test) but determined significantly (*p* = 0.021) lower DAI values in female *Alox5-*KI mice (Figure S4).

Taken together, our results suggest no significant differences in the responsiveness of male *Alox5*-KI mice and corresponding wildtype controls in the DSS colitis model, when the body weight loss was used as a clinical readout parameter. For female *Alox5*-KI mice, we observed a subtle but significantly lower colon shrinkage and significantly reduced DAI values. The limited protective effect we observed for our *Alox5*-KI mice in the DSS colitis model was somewhat surprising since the ALOX5 inhibitor zileuton protected animals in different models of inflammatory bowel disease [36,45]. A possible mechanistic explanation for this unexpected outcome is given in the discussion.

### 2.4. Alox5-KI Does Not Alter Mechanical and Heat Sensitivity in the Chronic Constriction Nerve Injury Model (CCI)

Pain is one of the five canonical symptoms of inflammation and ALOX isoforms have been implicated in the development of inflammatory pain [46,47,48]. ALOX inhibitors exhibited analgesic effects in different animal pain models such as somatic inflammatory pain [49,50], as well as back and neuropathic pain [36,51].

**Figure 7:**
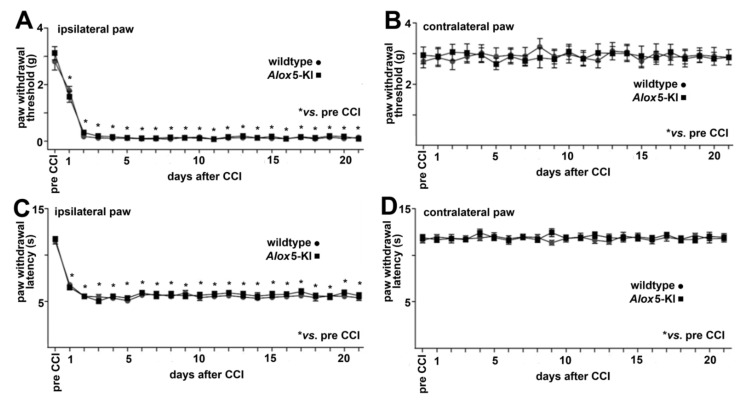
Comparison of *Alox5*-KI and wildtype mice in the CCI model of neuropathic pain. Mechanical sensitivity assessed by the von Frey test in paws ipsilateral (**A**) and contralateral (**B**) to the CCI. The decrease in the paw withdrawal threshold indicates mechanical hypersensitivity. Heat sensitivity assessed by the Hargreaves test in paws ipsilateral (**C**) and contralateral (**D**) to the CCI. The decrease in the paw withdrawal latency indicates heat hypersensitivity. * *p* < 0.001 vs. values before CCI induction (pre-CCI), one-way repeated measures ANOVA and Bonferroni’s multiple comparison test. Data are expressed as means ± SEM. *n* = 9 male mice per genotype.

In this study, we employed the CCI neuropathic pain model in which constriction of the sciatic nerve induces neuro-inflammation in the nerve and in the spinal cord, which leads to mechanical and heat hypersensitivity [52,53,54,55]. We found that both *Alox5*-KI and wildtype mice developed comparable CCI-triggered mechanical (manifested by decreased paw withdrawal thresholds assessed in the von Frey test) and heat hypersensitivity (manifested by shorter paw withdrawal latencies assessed by the Hargreaves test) in paws innervated by the injured nerve compared to the thresholds and latencies before CCI. Both forms of hypersensitivity appeared on day 1 of the experimental protocol and lasted until day 21 following CCI. There were no significant differences between the two genotypes when the experimental raw data were evaluated by one-way ANOVA (Figure 7A,C). We did not observe significant differences in the paws contralateral to the CCI in any test and genotype (Figure 7B,D). Taken together, these data suggests that our genetic manipulation of the *Alox5* gene does not modify the pain response in the CCI model.

## 3. Discussion

### 3.1. Alox5-KI Mice Are Leukotriene Deficient but Exhibit an Increased Capacity for 15-HETE Formation

Previous in vitro mutagenesis studies on recombinant ALOX5 orthologs of different vertebrates (humans, mice, zebrafish) indicated that combined mutations of the triad determinants induced a switch in reaction specificity from AA 5-lipoxygenation to AA 15-lipoxygenation [32,33,56]. To test whether a similar mutagenesis will also modify the reaction specificity of the mouse Alox5 ortholog in vivo, we created *Alox5*-KI mice, which express the Alox5 Phe359Trp + Ala424Ile + Asn425Met triple mutant instead of the wildtype enzyme [34]. Since the *Alox5* gene is predominantly expressed in white blood cells, we first performed ex vivo Alox5 activity assays using heparinized peripheral blood. For this purpose, we incubated total blood with calcium ionophore A23187 and quantified relevant eicosanoids (LTB_4_, 5-HETE, 15-HETE, 12-HETE, 9-HETE, 20-HETE) formed from endogenously released arachidonic acid. Our data indicate (Figure 2) that the *Alox5*-KI mice are leukotriene deficient but that the 15-HETE plasma levels are not significantly elevated in *Alox5*-KI mice. To obtain additional evidence for the leukotriene deficiency of our *Alox5*-KI mice we compared the pattern of selected reaction products formed from exogenously added and endogenously released AA by peritoneal lavage and bone marrow cells. Although both cell preparations are not homogenous but consist of different cell types, they are a reliable source for the catalytically active Alox5 protein and preparation of these cells is rather simple. When we characterized the major oxygenation products formed from exogenously added AA by these cell preparations, we observed attenuated formation of 5*S*-HETE but augmented formation of 15*S*-HETE (Figure 3). Similarly, when the AA cascade was initiated by the release of endogenous AA (stimulation of the cells with calcium ionophore) we found that the formation of 5-HETE and LTB_4_ was almost completely abolished. In contrast, endogenous 15-HETE formation was augmented (Figure 4). When similar activity assays were carried out in the presence of 10 μM zileuton formation of 5-HETE and LTB_4_ by wildtype cells was also completely abolished and 15-HETE formation by *Alox5*-KI cells was reduced to wildtype levels (Figures S1 and S2). Taken together, these data indicate that our genetic manipulations converted the wildtype Alox5 to an enzyme species that preferentially catalyzes AA 15-lipoxygenation. However, the sensitivity of the enzyme for the specific ALOX5 inhibitor zileuton was not altered.

### 3.2. Alox5-KI Mice and Alox5^−/−^ Animals Exhibit Differential Effects on EAE Score Kinetics

Leukotrienes, the major products of the ALOX5 pathway, are pro-inflammatory mediators [1] and thus interruption of their biosynthesis by systemic inactivation of the *Alox5* gene should induce anti-inflammatory effects. In fact, systemic *Alox5* deficiency reduced the degree of inflammatory symptoms in some but not in all peripheral inflammation models [16]. In neuro-inflammation, the biological roles of leukotrienes are less well documented. Antagonism at the leukotriene B4 receptor [57], dual ALOX5-COX inhibition [58] and prevention of leukotriene biosynthesis [40] attenuated EAE development, and these data are consistent with the assumption that systemic *Alox5* deficiency should protect mice form the development of EAE symptoms. In contrast, *Alox5*^−/−^ mice, which are unable to synthesize leukotrienes, developed more intense symptoms in the EAE model [35]. The mechanistic basis for this unexpected observation remains unclear but it might well be that in the central nervous system leukotrienes may mediate neuroprotective functions. When we tested our *Alox5*-KI mice, which also exhibit defective leukotriene biosynthesis, in the EAE model we also observed a trend to more intense neurological symptoms. Except for the cumulative incidence rate (Figure 5A), the observed differences did not reach the level of statistical significance (Figure 5B). To explain this outcome, one should keep in mind that our knock-in strategy did not only abolish the endogenous leukotriene biosynthesis [34] but it also upregulated the formation of 13-HODE [34] and 15-HETE (Figure 3, Figure 4). These compounds function as co-activators of PPARgamma [59,60]. Since PPARgamma activation has anti-inflammatory effects, improvement of the clinical outcome was expected. In other words, the first consequence of our knock-in strategy (prevention of endogenous leukotriene biosynthesis), which leads to exacerbation of the EAE symptoms, is counteracted by the second consequence (augmented biosynthesis of 13-HODE and 15-HETE), which should reduce inflammatory symptoms. This mechanistic scenario is consistent with our experimental observation (Figure 5B), but still does not explain why *Alox5*^−/−^ mice are more sensitive than wildtype controls in the EAE model [35].

### 3.3. Alox5-KI Mice in the DSS Colitis Model

Because of its key role in the biosynthesis of inflammatory leukotrienes, ALOX5 is considered a pro-inflammatory enzyme [16]. Experiments with the specific ALOX5 inhibitor (zileuton) in different murine colitis models have indicated that interference with endogenous leukotriene biosynthesis protected the animals from colitis development [36,45]. Treatment of rats with zileuton also improved the outcome of intestinal inflammation, and the degree of the protective effect was comparable with that induced by systemic application of the frequently used specific COX2 inhibitor celecoxib [61]. Since our *Alox5*-KI mice were unable to biosynthesize leukotrienes [34], it was expected that these animals should also be protected from DSS-induced colitis. However, our data (Figure 6C,D, Figure S4) indicate that male mice were not protected in the DSS colitis model. For most clinical readout parameters of colitis severity (body weight kinetics, colon shortening, DAI), we did not observe significant differences between *Alox5*-KI mice and wildtype controls. For females, we did not observe significant differences in the body weight kinetics (Figure 6A), but did detect subtle but significant differences in the degree of colon shortening (Figure 6B) and the DAI (Figure S4). However, these differences are clearly less pronounced than those observed in the pharmacological intervention studies using the ALOX5 inhibitor zileuton [36,45,61]. For the time being, the molecular basis for the observation that our *Alox5*-KI mice behave differently than *Alox5*^−/−^ mice has not been explored. It should, however, be stressed that our genetic manipulation not only induced dysfunctional leukotriene biosynthesis [34] but also upregulated the formation of the ALOX15 products 15-HETE (Figure 3A vs. Figure 3B, Figure 4E vs. Figure 4F) and 13-HODE [34]. In DSS colitis, systemic inactivation of the *Alox15* gene (*Alox15*^−/−^ mice) protected female mice from the development of colitis symptoms and transgenic expression of human ALOX15 under the control of the aP2 promoter deteriorated the outcome of the disease [28]. In other words, in DSS colitis the first effect of our genetic manipulation (prevention of endogenous leukotriene biosynthesis) is likely to induce less severe colitis symptoms but this protective effect might be compensated by the augmented formation of Alox15 products by the mutant enzyme.

### 3.4. Alox5 and Neuropathic Pain

Previous experiments suggested that ALOX inhibitors exhibited analgesic effects in different animal pain models, such as somatic inflammatory pain [49,50] as well as back and neuropathic pain [36,51]. Here we explored whether genetically modified mice (*Alox5*-KI mice) expressing an Alox5 variant that is not capable of synthesizing pro-inflammatory leukotrienes [34] are protected from pain development in the CCI model. Interestingly, we found that these mice (Figure 7) were not protected, and this data suggests that the formation of leukotrienes may not play a major patho-physiological role in the CCI pain model. Unfortunately, to the best of our knowledge *Alox5*^−/−^ mice have not been tested in this particular pain model, and thus the two genotypes (*Alox5* knock-out vs. *Alox5* knock-in) cannot directly be compared.

## 4. Materials and Methods

### 4.1. Chemicals

The chemicals used for the characterization experiments were obtained from the following sources: 5,8,11,14-all-*cis*-eicosatetraenoic acid (arachidonic acid, AA), HPLC standards of 5S/R-HETE, 12S/R-HETE, 15S/R-HETE, 5S-HETE, 12S-HETE, 15S-HETE from Cayman Chem (distributed by Biomol, Hamburg, Germany); HPLC solvents from Fisher Scientific (Schwerte, Germany). The sources of the chemicals used in the animal disease models are specified in the description of the model systems.

### 4.2. Genetically Modified Animals and Genotyping

All mice were bred and maintained in a specific pathogen-free (SPF) animal facility, on 12h:12h LD cycle according to the FELASA recommendation with food and water ad libitum. These experiments were approved by the State animal care committee (Landesamt für Gesundheit und Soziales, Berlin, Germany) and were performed according to the Guidelines for the Care and Use of Laboratory Animals adopted by the U.S. National Institutes of Health, and the ARRIVE guidelines. The following permission numbers were given: DSS colitis model (G0250/15), EAE encephalomyelitis model (G0053/12, G0085/16), CCI neuropathic pain model (G 0017/15).

All animals included in this study were independently genotyped for the *Alox5* [34] gene locus. *Alox5* knock-in mice (*Alox5*-KI), which express the Alox5 triple mutant (Phe359Trp + Ala424Ile + Asn425Met) instead of the wildtype enzyme, were created by homologous recombination of the mutant gene construct into the genome of mouse embryonic stem cells. These mice were viable, breed normally and did not show major phenotypic defects. The mice were also backcrossed into C57BL/6 background and C57BL/6 wildtype mice were used as corresponding controls.

### 4.3. Cell Preparation for Functional Characterization

To isolate bone marrow cells, the skin was removed from the hind limbs of sacrificed mice and femurs were carefully cleaned from adherent soft tissue. The *acetabulum* was dislocated from the hip joint and the femur was separated from the tibia at the knee joint. Both ends of each femur were cut off using a scalpel and the bone marrow cells were prepared by rinsing the bone marrow cavity of the femur with 1 mL PBS. Cells were washed once with 1 mL of PBS, pelleted by centrifugation (800× *g*) and reconstituted in 0.5 mL of PBS. Aliquots of this suspension were used for cellular ALOX activity assays and for RNA preparation. To prepare peritoneal lavage cells, the skin was removed from the belly region of sacrificed mice and 10 mL of PBS were injected into the peritoneal cavity. Care was taken not to injure organs in the peritoneal cavity. After a 5 min massage period the majority of the peritoneal fluid was removed by aspiration. Subsequently, the peritoneal cavity was opened, and remaining fluid was aspirated. Cells were spun down, washed twice with PBS and reconstituted in 0.5 mL PBS. Aliquots of this suspension were used for ex vivo Alox activity assays.

### 4.4. RNA Extraction and qRT-PCR of the Different Alox-Isoforms

Peritoneal macrophages or bone marrow cells prepared from each individual were disrupted in Lysing matrix D tubes containing 600 μL LBP (NucleoSpin kit) with a FastPrep 24 homogenizer (MP Biomedicals GmbH, Eschwege, Germany; 3 × 40 s). Debris was spun down, the supernatant was recovered and cleared using Qia-shredder columns (Qiagen GmbH, Hilden, Germany). Total RNA was prepared from the eluate using the NucleoSpin RNA plus extraction kit (Macherey-Nagel, Düren, Germany) according to the manufacturer’s instructions. The corresponding cDNA was synthesized by reverse transcription of 0.2–0.5 μg of total RNA using Tetro Reverse Transcriptase (Bioline, Luckenwalde, Germany, distributed by BioCat GmbH Heidelberg) and Oligo-dT18 primers. External amplification standards for each gene were cloned into the TOPO 2.1 vector (Invitrogen-Thermo Fisher Scientific, Dreieich, Germany) and the linearized vector with known ssDNA copy number/μL was used as calibration standard for the PCR products. With these calibration curves, we quantified the number of mRNA copies of target genes in the total RNA extract. qRT-PCR (SensiFastSybr no-Rox Kit; Bioline, distributed by BioCat GmbH Heidelberg) was performed on a Rotor-Gene RG 3000 (Corbett Research Ltd., Saffron Walden, UK) started with a denaturation step (10 min at 95 °C). Afterwards, 40 cycles of amplification, each consisting of a denaturation (15 s at 95 °C), an annealing (30 s at 65 °C), and a synthesis period (20 s at 72 °C) were carried out. A housekeeping gene *mGapdh* was used. The gene specific amplification primers listed in Table 2 were used (BioTez Berlin-Buch GmbH, Germany).

### 4.5. Cellular Ex Vivo Arachidonic Acid Oxygenase Activity Assays Using Exogenous Substrates

To assay the cellular arachidonic acid oxygenase activity, the bone marrow cells and/or the peritoneal lavage cells prepared from a single individual were incubated in 1 mL of PBS for 15 min in the presence of 80 μM arachidonic acid (AA). To optimize the Alox5 activity we added 100 μM ATP, 100 μM EDTA, 0.4 mM Ca^2+^ and 1.4 μg/mL soybean phosphatidylcholine to the activity assay mixture. After a 15 min incubation period, the hydroperoxy fatty acids formed were reduced by the addition of 1 mg solid sodium borohydride, the samples were acidified and extracted twice with 1 mL of ethyl acetate. The extracts were combined, the solvent was evaporated, the extracted lipids were reconstituted in 0.5 mL of HPLC column solvent (acetonitrile/water/acetic acid = 70/30/0.1, by vol) and analyzed by RP-HPLC on a Shimadzu LC-20 instrument equipped with a SIL-20AC autoinjector, a SPD-M20A diode array detector and a CTO-20AC column oven. The oxygenated fatty acids were analyzed on a Nucleodur 100-5 C_18ec_ column, which was connected with a corresponding 1 cm guard column. Isocratic elution at 25 °C was carried out. Metabolites were identified by the retention times and their UV-spectral properties.

Since RP-HPLC does not separate HETE enantiomers, we applied an alternative methodological approach to obtain information on the enantiomer composition of the ALOX products formed. For this purpose, the conjugated dienes formed were prepared by RP-HPLC (see legend to Figure 1) and further analyzed by a combination of normal phase and chiral phase HPLC (NP/CP-HPLC). NP-HPLC guard column Nucleosil (Nucleosil 50–5, 4.6 mm x 35 mm, 5 μm particle size, Macherey-Nagel, Düren, Germany) was set in front of a CP-HPLC column (Chiralpak AD-H, 4.6 mm × 250 mm, 5 μm particle size, Daicel, Osaka, Japan) and the RP-HPLC purified analytes were isocratically eluted with the solvent system n-hexane/methanol/ethanol/acetic acid (96:3:1:0.1, by vol) at a flow rate of 1 mL/min at room temperature.

### 4.6. Ex Vivo Alox5 Activity Assays Using Heparinized Whole Peripheral Blood

For ex vivo Alox5 activity assays, heparinized blood was collected from *Alox5*-KI mice and outbred WT controls. In total, 0.5 mL of blood was incubated for 30 min at 37 °C in the presence of 50 μM calcium ionophore (A23187). Blood cells were spun down, the plasma was recovered and was analyzed for relevant eicosanoids (LTB4, 5-HETE, 12-HETE, 15-HETE, 9-HETE, 20-HETE) by LC-MS/MS. In detail, 50 μL plasma was spiked with external deuterated standards (14,15-EET-d8, 14,15-DHET-D11, 15-HETE-d8, 20-HETE-d6, LTB4-d4, PGE2-d2, 0.1 ng each, Cayman Chem., distributed by Biomol, Hamburg, Germany). Then 450 μL water, 20 μL glycerol, 500 μL acetonitrile and 5 μL 2,6-Di-tert-butyl-4-methylphenol (BHT 10 mg/mL) were added to the blood plasma. Then the pH was adjusted to 6 by addition of 2 mL phosphate buffer (0.1 mol/L). Protein precipitate was removed by centrifugation and the obtained supernatant was loaded onto Bond Elute Certify II columns (Agilent Technologies) for solid-phase lipid extraction. For this purpose, the columns were preconditioned by eluting them sequentially with 3 mL methanol and then with 3 mL of 0.1 mol/L phosphate buffer (pH 6) containing 5% methanol. After sample application the columns were washed with 3 mL methanol/water (40/50, by vol) and the metabolites were eluted with 2 mL of a mixture of n-hexane: ethyl acetate (25: 75 containing 1% acetic acid). The solvents were evaporated on a heating block at 40 °C under a stream of nitrogen and the remaining lipids were reconstituted in 100 μL of a methanol/water mixture (60:40, by vol).

The samples were analyzed using an Agilent 1290 HPLC system (Agilent, Waldbronn, Germany) with binary pump, multisampler and column thermostat using an Agilent Zorbax Eclipse plus C-18 column (2.1 × 150 mm, 1.8 μm particle size) with a solvent system consisting of aqueous acetic acid (0.05%) and acetonitrile. The elution gradient was started with 5% acetonitrile, which was increased within 0.5 min to 32%, 16 min to 36.5%, 20 min to 38%, 28 min to 98% and held there for 5 min. The flow rate was set at 0.3 mL/min, the injection volume was 20 μL. The HPLC was coupled with an Agilent 6495 Triplequad mass spectrometer (Agilent, Waldbronn, Germany) with electrospray ionisation source and the following source parameters were employed: drying gas (115 °C/16 L/min), sheath gas (390 °C/12 L/min), capillary voltage (4300 V), nozzel voltage (1950 V) and Nebulizer pressure (35 psi). Analysis was carried out using Multiple Reaction Monitoring in negative mode. The GraphPad Prism software version 8.0.0 for iOS was used to structure Figure 2 (GraphPad Software, San Diego, CA, USA, www.graphpad.com, accessed on 26 July 2021).

### 4.7. Ex Vivo Formation of LTB4, 5-HETE and 15-HETE by Isolated Bone Marrow Cells Using Endogenous AA as Substrate

To explore whether the mutagenesis induced alterations in the cellular eicosanoid metabolism (abolished 5-HETE and LTB_4_ formation and increased formation of 15-HETE) can be confirmed when endogenous substrates were used, we isolated bone marrow cells from female *Alox5*-KI mice and corresponding WT controls. Cells were washed and then reconstituted in PBS reaching a final cell concentration of 4 × 10^−7^ per mL. In total, 0.5 mL of these cellular stock solutions (2 × 10^−7^ cells) were taken and used for the activity assays. The ALOX reaction was initiated by the addition of 5 μM calcium ionophore A23187 and the cells were incubated for 5 min at room temperature. After the incubation period the sample was acidified by the addition of glacial acetic acid and the total lipids were extracted twice with 1 mL of ethyl acetate. The extracts were pooled, the solvent was evaporated, and the remaining lipids were reconstituted in 0.05 mL of methanol and analyzed for the formation of 5-HETE, 15-HETE and LTB_4_ by LC-MS/MS. For some experiments the cells were pretreated with the specific ALOX5 inhibitor zileuton before the Alox reaction was initiated. In these cases, cells (0.5 mL involving 2 × 10^−7^ cells) were pre-incubated in the presence of 10 μM zileuton at 37 °C for 20 min. After pre-incubation, cells were spun down, washed once with PBS and reconstituted in 0.5 mL of PBS. Then the Alox reaction with endogenous substrate was initiated by addition of calcium ionophore.

For LC-MS/MS analysis the lipid extracts were spiked with deuterated internal standards including ^2^H_4_-LTB4,^2^H_4_-9-HODE, ^2^H_8_-5-HETE, ^2^H_8_-12-HETE and ^2^H_6_-20-HETE, and quantification of the Alox products was carried out by LC-MS/MS [37]. In brief, chromatographic separation of the reaction products (injection volume 5–10 μL) was carried out on an Agilent LC instrument (Agilent, Waldbronn, Germany) equipped with an Agilent Zorbax Eclipse Plus C18 reversed phase column (2.1 × 150 mm, particle size 1.8 μm). A binary solvent gradient with 0.1% aqueous acetic acid as solvent A and acetonitrile/methanol/acetic acid (800/150/1, *v*/*v*/*v*) as solvent B at a flow rate of 0.3 mL/min was used. Mass spectrometric detection was performed on an AB Sciex 6500 QTRAP instrument (SCIEX, Darmstadt, Germany) in the selected reaction monitoring mode following negative electrospray ionization. Data analysis was carried out with Multiquant 2.1.1. software package.

### 4.8. Experimental Autoimmune Encephalomyelitis (EAE) Model

To induce active EAE, 12-week-old female wildtype and *Alox5*-KI mice were immunized subcutaneously with 200 μL of myelin oligodendrocyte glycoprotein peptide 35–55 (MOG35-55) (Pepceuticals, Leicester, UK) emulsified in complete Freund’s adjuvant (Difco Laboratories, Detroit, MI, USA) containing 800 μg Mycobacterium tuberculosis H37Ra (Difco). At the day of immunization and 48 h later, mice were intraperitoneally injected with Pertussis toxin (200 ng, Sigma-Aldrich, St. Louis, MO, USA). Clinical symptoms were monitored daily and scored as follows: 0, no symptoms; 0.5, tail weakness; 1, lack of tail tone; 1.5, no righting reflex; 2, hind-limb weakness; 2.5, partial hind-limb paralysis; 3, total hind-limb paralysis; 3.5, ascending fore-limb paralysis. Statistical analyses of the experimental raw data obtained were performed using the SPSS software packages. Cumulative incidences were statistically analyzed by the Log-Rank test (Figure 4A). Time-course data (Figure 4B) between two genotypes were compared by mixed analysis of variance (ANOVA) using the Greenhouse-Geisser correction. The normalized spleen weight data (Figure 4C) for the two genotypes were evaluated with the Mann-Whitney U-test. Differences were considered significant at values of *p* < 0.05.

### 4.9. Dextran Sodium Sulfate (DSS)-Induced Colitis Model

For induction of colitis, 12-week-old wildtype (5 males and 5 females) and *Alox5*-KI (5 males and 5 females) mice received 2.5% (wt/vol) dextran sodium sulfate (DSS; molecular weight = 36,000–50,000; MP Biomedicals, Eschwege, Germany) in the drinking water for 6 days followed by 3 days of normal drinking water. On the 9th day mice were sacrificed by isoflurane anesthesia and cervical dislocation. The colon was prepared, colon length was determined, and colonic tissue was snap frozen.

Statistical analyses of the experimental raw data obtained in this series of experiments were performed using the SPSS Statistics software packages. Comparison of two groups were performed using Mann-Whitney U test. The time-course data of the two genotypes were compared by mixed analysis of variance (ANOVA, using Greenhouse-Geisser correction). Differences were considered significant at values of *p* < 0.05.

### 4.10. Chronic Constriction Injury (CCI) Model of Neuropathic Pain

*Alox*5-KI mice and corresponding wildtype controls (26–32 g, 10–11 weeks old; bred at the Charité-Campus Benjamin Franklin, Berlin, Germany) were kept in groups of 3 per cage, with free access to food and water, in environmentally controlled conditions (12 h light/dark schedule, light on at 7:00 h; 22–24 °C; humidity 60–65%). The experiments were performed by an experimenter blinded to the genotypes. The codes were broken after completion of the experiments. After the experiments the animals were killed with an overdose of isoflurane.

CCI was induced in deeply isoflurane-anesthetized mice. The sciatic nerve was exposed at the level of the right mid-thigh and three loose silk ligatures (4/0) were placed around the nerve with 1 mm spacing. The ligatures were tied until they elicited a brief twitch in the right hind limb. The wound was closed with silk sutures [54,55,62].

Mechanical sensitivity was assessed using the von Frey test. For this purpose, the animals were habituated to the test cages daily (1–2 times for 15 min), starting 6 days prior to nociceptive testing; they were individually placed in clear Plexiglas chambers located on a stand with anodized mesh (Model 410; IITC Life Sciences, Woodland Hills, Los Angeles, CA, USA). To assess the sensitivity, calibrated von Frey filaments in the range of 0.054 mN (0.0056 g) to 42.85 mN (4.37 g) were used. The filaments were applied until they bowed, for approximately 3 s, to the plantar surface of hind paws. The up-down method was used to estimate 50% withdrawal thresholds [63]. Testing was started using a 2.74 mN (0.28 g) filament. If the animal withdrew the paw, the preceding weaker filament was applied. In the absence of withdrawal, the next stronger filament was applied. The maximum number of applications was 6–9, and the cut-off was 42.85 mN (4.37 g) according to our previous studies [54,55,62].

Heat sensitivity was assessed using the Hargreaves test. Mice were habituated to the test cages daily (1–2 times for 15 min), starting 6 days prior to nociceptive testing; they were placed in clear Plexiglas chambers positioned on a stand with a glass surface (Model 336; IITC Life Sciences, Woodland Hills, Los Angeles, CA, USA). To examine heat sensitivity, radiant heat was applied to the plantar surface of hind paws from underneath the glass floor with a high intensity projector lamp bulb and paw withdrawal latency was evaluated using an electronic timer. The withdrawal latency was defined as the average of two measurements separated by at least 10 s. The heat intensity was adjusted to obtain baseline withdrawal latency of about 10–12 s in uninjured paws, and the cut-off was set at 20 s to avoid tissue damage [55,62,64]. Mechanical and heat sensitivity were evaluated in the same groups of mice with an interval of at least one hour between the tests, a day before and then daily on days 1–21 after CCI. Nine wildtype and nine *Alox5*-KI male mice were used. Statistical analyses of the experimental raw data obtained in this series of experiments were performed using GraphPad Prism software (Version 5.02 for Windows; GraphPad Software Inc.). Two groups over time were compared by two-way repeated measures analysis of variance (ANOVA). Multiple comparisons over time within one group were performed by one-way repeated measures ANOVA followed by Bonferroni’s multiple comparison test. Differences were considered significant at values of *p* < 0.05.

## 5. Conclusions

As *Alox5* knock-out mice (*Alox5^−/−^)*, our *Alox5* knock-in mice (*Alox5-KI* mice) expressing an arachidonic acid 15-lipoxygenating Alox5 mutant were leukotriene deficient. However, in the EAE disease model the two genotypes exhibited differential effects, which might be related to the increased capability of the mutant Alox5 to synthesize 15-H(p)ETE and/or 15-H(p)ETE derived secondary products. In two other animal inflammation models (DSS colitis model, CCI model of neuropathic pain) the *Alox5-KI* mice behaved like wildtype control animals.

## Figures and Tables

**Table 1:** Composition of the major oxygenation products formed during the ex vivo activity assays using different cell preparations from *Alox5*-KI mice and wildtype controls. Cell preparation, activity assays and RP-HPLC analyses were carried out as described in the legend to Figure 2. The sum of the major oxygenation products was set at 100% and the relative shares of 15-HETE, 12-HETE and 5-HETE were calculated. Four different animals (*n* = 4) were analyzed for each genotype. To quantify the extent of product formation we subtracted the sizes of the chromatographic peaks (area units) of the no-cell control incubations from the corresponding peak of the cell incubations. In some cases, we found that the 5-HETE levels in the cell incubations were smaller than those in the no-cell control incubations. In these cases, we set the differences to 0. * Comparison (two-sided *t*-test) of the relative shares of each product (12*S*-HETE, 15*S*-HETE, 5*S*-HETE) between *Alox5*-KI animals and wildtype controls revealed highly significant (*p* < 0.01) differences. ^#^ The difference in 12*S*-HETE formation by peritoneal lavage cells (84.1 ± 2.9% 12*S*-HETE formation for wildtype cells vs. 82.4 ± 2.4% 12*S*-HETE formation for *Alox5*-KI cells, *p* = 0.316) did not reach the level of statistical significance.

Cell Type	Genotype	Product (%)		
		5*S*-HETE	12*S*-HETE	15*S*-HETE
Peritoneal lavage cells	WT	7.8 ± 2.7	84.1 ± 2.9	8.1 ± 0.4
	*Alox5*-KI	0 *	82.4 ± 2.4 ^#^	18.0 ± 2.4 *
Bone marrow cells	WT	12.0 ± 1.4	85.8 ± 1.8	2.2 ± 0.5
	*Alox5*-KI	0 *	89.7 ± 0.4 *	10.3 ± 0.4 *

**Table 2:** Sequence of the gene specific amplification primers used for qRT-PCR.

Gene	Forward Primer 5′→ 3′	Reverse Primer 5′ → 3′
*mGapdh*	CCATCACCATCTTCCAGAGCGA	GGATGACCTTGCCCACAGCCTTG
*mAlox15*	GTACGCGGGCTCCAACAACGA	TCTCCGGGGCCCTTCACAGAA
*mAlox15b*	CCTCCCGCTTATGTCTTTCCGT	GCCCTTTGACTTTCAGCTCCGTA
*mAlox12*	GCGGCCATGTTCAGTTGCTTAC	CATCGTCACGTCGTCCTTGCTG
*mAlox5*	TCGAGTTCCCATGTTACCGCT	CTGTGGTCACTGGGAGCTTCG
*mAlox12b*	GGTGATGGTTCGGGGTCTGTCT	GAGTCCAGAGCACCAAGAGCACA
*mAloxe12*	CTCCAGCCACCACGACACGG	GCAACGAGTCCACAATGTCCCT
*mAloxe3*	GGGCGGCTATTGAGAGGTTTGT	TCTGGTCCTTTGGCTCTTGGCT

## Data Availability

The data presented in this paper is available on request from the corresponding author. The original experimental raw data are not publicly available. They are kept by the researchers who carried out the different experiments (see Authors Contribution) but will be send on request to interested scientists.

## Supplementary Materials

The following items are available online at https://www.mdpi.com/article/10.3390/metabo11100698/s1: Figure S1: Impact of the ALOX5 specific inhibitor zileuton on 5-HETE formation of bone marrow cells from endogenous arachidonic acid, Figure S2: Impact of the ALOX5 specific inhibitor zileuton on 15-HETE formation of bone marrow cells from endogenous arachidonic acid, Figure S3: Comparison of different lymphocyte populations in the peripheral blood of *Alox5*-KI mice and corresponding wildtype control animals at the end (day 22) of the experimental protocol of the EAE model, Figure S4: Disease activity index of male and female mice at the end (day 9) of the DSS-induced experimental colitis.
