# Supplementary material for: Knock-In Mice Expressing a 15-Lipoxygenating Alox5 Mutant Respond Differently to Experimental Inflammation Than Reported Alox5−/− Mice
Source: Metabolites. 2021 Oct 12;11(10):698. doi: 10.3390/metabo11100698 (PMC8538363; doi:10.3390/metabo11100698)
Supplement: Supplementary file 1 [file metabolites-11-00698-s001.zip › metabolites-1336317-supplementary.pdf]

*metabolites*

*Supplementary Material*

# Knock-In Mice Expressing a 15-Lipoxygenating Alox5 Mutant Respond Differently to Experimental Inflammation than Reported *Alox5*$^{-/-}$ Mice

**Eugenia Marbach-Breitrück [1], Nadine Rohwer [2,3,4,5], Carmen Infante-Duarte [6], Silvina Romero-Suarez [6], Dominika Labuz [7], Halina Machelska [7], Laura Kutzner [8], Nils Helge Schebb [8], Michael Rothe [9], Pallu Reddanna [10], Karsten H. Weylandt [2,3,4,5], Lothar H. Wieler [11,12], Dagmar Heydeck [1] and Hartmut Kuhn [1,*]**

1 Charité-Universitätsmedizin Berlin, corporate member of Freie Universität Berlin and Humboldt-Universität zu Berlin, Department of Biochemistry, Chariteplatz 1, D-10117 Berlin, Germany; eugenia.marbach@yahoo.de (E.M.-B.); dagmar.heydeck@charite.de (D.H.)
2 Division of Medicine, Department of Gastroenterology, Metabolism and Oncology, Ruppin General Hospital, Brandenburg Medical School, Fehrbelliner Straße 38, 16816 Neuruppin, Germany; nadine.rohwer@mhb-fontane.de (N.R.); karsten.weylandt@mhb-fontane.de (K.H.W.)
3 Faculty of Health Sciences, Joint Faculty of the Brandenburg University of Technology Cottbus–Senftenberg, Brandenburg Medical School and University of Potsdam, 14469 Potsdam, Germany
4 Charité-Universitätsmedizin Berlin, Corporate member of Freie Universität Berlin and Humboldt-Universität zu Berlin, Department of Hepatology, Gastroenterology and Metabolism, Augustenburger Platz 1, 13353 Berlin, Germany
5 Department of Molecular Toxicology, German Institute of Human Nutrition Potsdam-Rehbruecke, Arthur-Scheunert-Allee 114-116, 14558 Nuthetal, Germany
6 Charité-Universitätsmedizin Berlin, Corporate member of Freie Universität Berlin and Humboldt-Universität zu Berlin, Institute for Medical Immunology, Augustenburger Platz 1, 13353 Berlin, Germany; carmen.infante@charite.de (C.I.-D.); silvina.romero-suarez@medma.uni-heidelberg.de (S.R.-S.)
7 Charité-Universitätsmedizin Berlin, Corporate member of Freie Universität Berlin and Humboldt-Universität zu Berlin, Department of Experimental Anesthesiology, Hindenburgdamm 30, 12203 Berlin; dominika.labuz@charite.de (D.L.); hmachelska@gmail.com (H.M.)
8 Chair of Food Chemistry, Faculty of Mathematics and Natural Sciences, University of Wuppertal, Gaussstraße 20, 42119 Wuppertal, Germany; laura.kutzner@schebb-web.de (L.K.); nils@schebb-web.de (N.H.S.)
9 Lipidomix GmbH, Robert-Rössle-Straße 10, 13125 Berlin, Germany; michael.rothe@lipidomix.de
10 Department of Animal Biology, School of Life Sciences, University of Hyderabad, Gachibowli, Hyderabad 500046, Andhra Pradesh, India; preddanna@gmail.com
11 Robert Koch Institute, Nordufer 20, 13353 Berlin, Germany; WielerLH@rki.de
12 Center of Infection Medicine, Institute of Microbiology and Epizootics, Free University of Berlin, Robert-von-Ostertag-Str. 7-13, 14163 Berlin, Germany;
* Correspondence: hartmut.kuehn@charite.de

**Citation:** Marbach-Breitrück, E.; Rohwer, N.; Infante-Duarte, C.; Romero-Suarez, S.; Labuz, D.; Machelska, H.; Kutzner, L.; Schebb, N.H.; Reddanna, P.; Weylandt, K.H.; et al. Knock-In Mice Expressing a 15-Lipoxygenating Alox5 Mutant Respond Differently to Experimental Inflammation Than Reported Alox5$^{-/-}$ Mice. *Metabolites* **2021**, *11*, 698. https://doi.org/10.3390/metabo11100698

Academic Editor: Helena Idborg

Received: 27 July 2021
Accepted: 7 October 2021
Published: 12 October 2021

**Publisher's Note:** MDPI stays neutral with regard to jurisdictional claims in published maps and institutional affiliations.

**Copyright:** © 2021 by the authors. Licensee MDPI, Basel, Switzerland. This article is an open access article distributed under the terms and conditions of the Creative Commons Attribution (CC BY) license (http://creativecommons.org/licenses/by/4.0/).

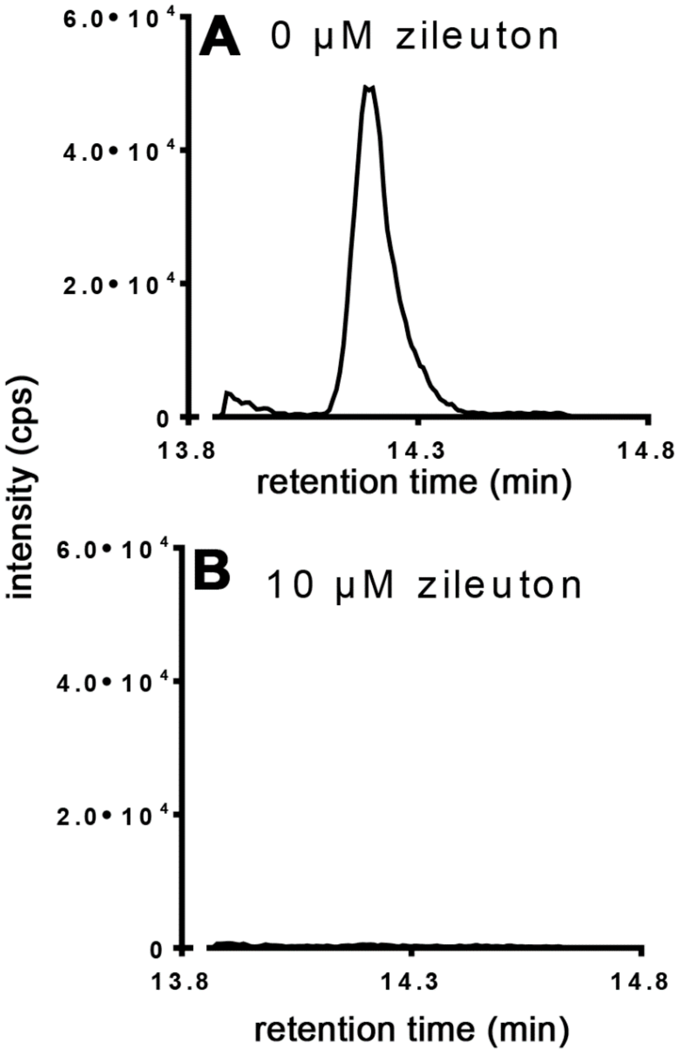


**Figure S1.** Impact of the ALOX5 specific inhibitor zileuton on 5-HETE formation of bone marrow cells from endogenous arachidonic acid. Pooled bone marrow cells of WT-mice were used. For each assay $2x10^7$cells were reconstituted in 0.5 ml PBS. To the zileuton sample 10 µM of the inhibitor (0.5 µL of a 10 mM stock solution in DMSO) was added. To the no zileuton sample 0.5 µL of DMSO was supplied and this sample was used as solvent control. The cell suspensions were pre-incubated in the presence and absence of zileuton for 5 min. Then 5 µM calcium ionophore A23187 was added and the cells suspensions were further incubated for 5 min. Total lipids were extracted with ethyl acetate, the solvent was evaporated, the remaining lipids were reconstituted in 50 µL methanol and this solution was analyzed for $LTB_4$ by LC-MS/MS. Chromatographic separation of lipid extracts was carried out using a Zorbax Eclipse Plus C18 RP-column (Agilent) and for MS/MS detection the QTRAP instrument (Sciex) was operated in negative electrospray ionization mode. Shown are SRM traces of the lipid extracts for $LTB_4$ (*m/z* 335.2 → 195.1) A) Cell incubation in the absence of zileuton, B) cell incubation in the presence of 10 µM zileuton.

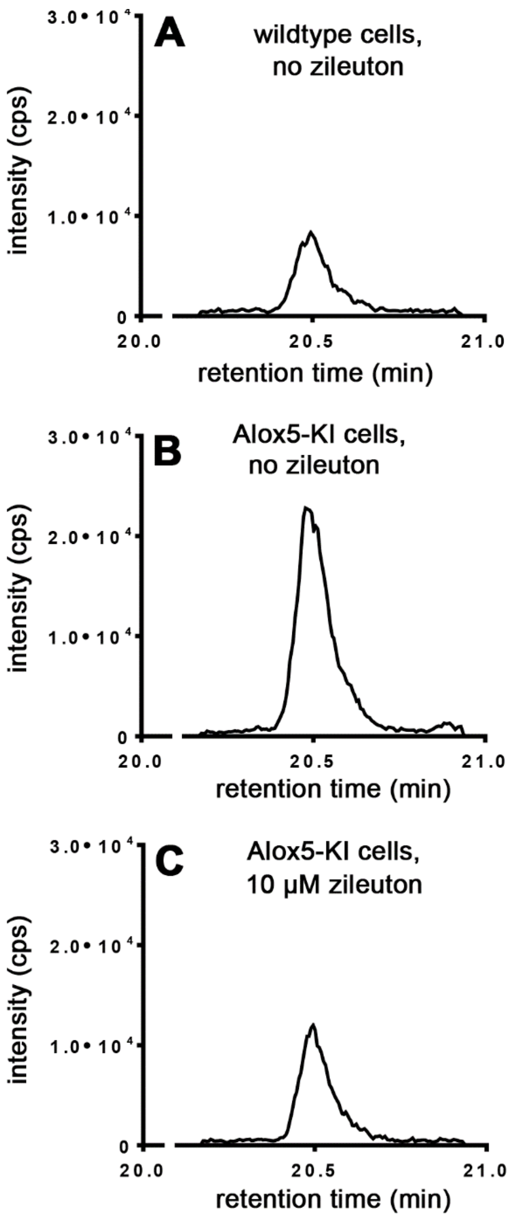


**Figure S2.** Impact of the ALOX5 specific inhibitor zileuton on 15-HETE formation of bone marrow cells from endogenous arachidonic acid. Pooled bone marrow cells of WT-mice and *Alox5*-KI mice were used. For each assay $2x10^7$cells were reconstituted in 0.5 ml PBS. To the zileuton sample 10 µM of the inhibitor (0.5 µL of a 10 mM stock solution in DMSO) was added. To the no zileuton samples 0.5 µL of DMSO was supplied and these samples were used as solvent control. The cell suspensions were pre-incubated in the presence and absence of zileuton for 5 min. Then 5 µM calcium ionophore A23187 was added and the suspensions were further incubated for 5 min. Total lipids were extracted with ethyl acetate, the solvent was evaporated, the remaining lipids were reconstituted in 50 µL methanol and this solution was analyzed for 15-HETE by LC-MS/MS. Chromatographic separation of lipid extracts was carried out using a Zorbax Eclipse Plus C18 RP-column (Agilent) and for MS/MS detection the QTRAP instrument (Sciex) was operated in negative electrospray ionisation mode. Shown are SRM traces of cell extracts for 15-HETE (m/z 319.2 → 219.2).

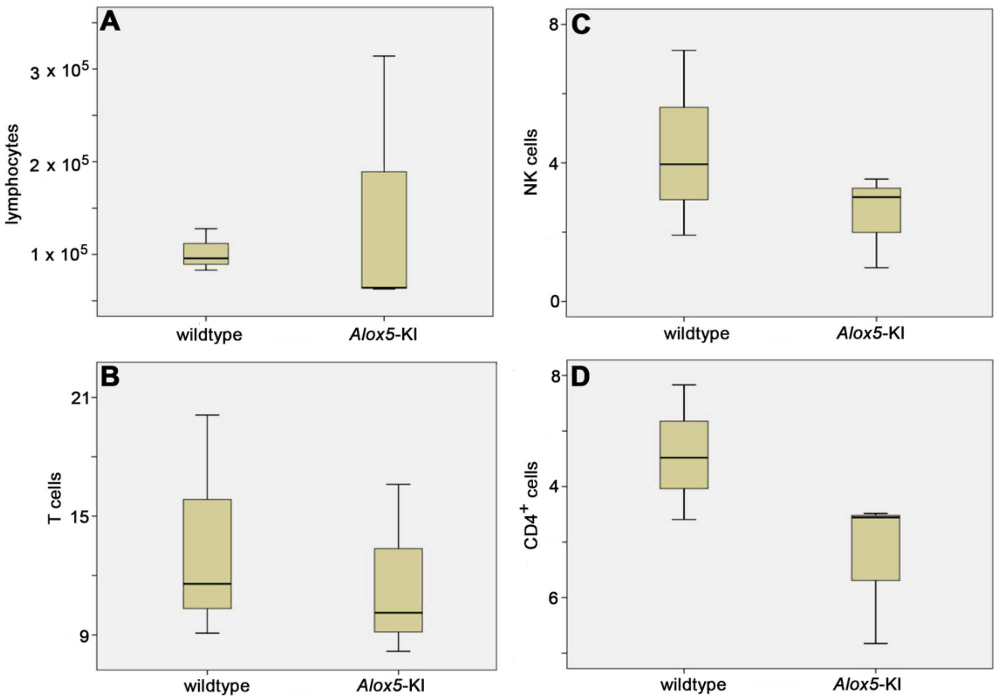


**Figure S3.** Comparison of different lymphocyte populations in the peripheral blood of *Alox5*-KI mice and corresponding wildtype controls mice at the end (day 22) of the experimental protocol of the EAE model (Figure 4). *Alox5*-KI mice and corresponding wildtype controls (n=8 in each group) were taken through the experimental protocol (see Materials and Methods). Blood was removed from three animals of each experimental group and the different types of lymphocyte were quantified. Experimental raw data are shown as box-plots and were statistically evaluated with the two-sided t-test using the SPSS software package. A) Total number of lymphocyte, B) Total numbers of T cells, C) Total numbers of natural killer cells (NK), D) Total number of CD4$^{+}$ T cells.

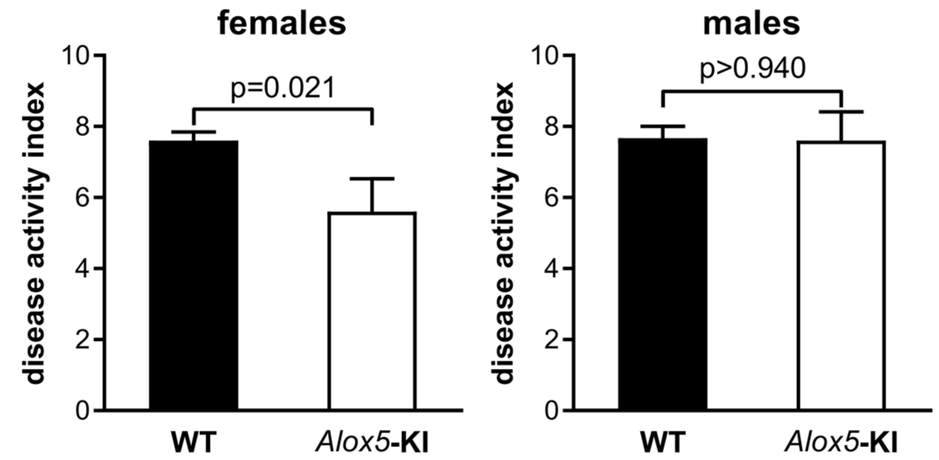


***Figure S4.*** Disease activity index of male and female mice at the end (day 9) of the DSS-induced experimental colitis. *Alox5*-KI mice (5 males, 5 females) and outbred wildtype controls (5 males, 5 females) were taken through the experimental protocol (see Materials and Methods) and the disease activity index ws determined. This value characterizes the severity of the inflammatory alterations [1].

1. Eichele, D.D.; Kharbanda, K.K. Dextran sodium sulfate colitis murine model: An indispensable tool for advancing our understanding of inflammatory bowel diseases pathogenesis. *World journal of gastroenterology : WJG* **2017**, *23*, 6016-6029, doi:10.3748/wjg.v23.i33.6016.
